# DARC 2.0: Improved Docking and Virtual Screening at Protein Interaction Sites

**DOI:** 10.1371/journal.pone.0131612

**Published:** 2015-07-16

**Authors:** Ragul Gowthaman, Sergey Lyskov, John Karanicolas

**Affiliations:** 1 Center for Computational Biology, University of Kansas, 2030 Becker Dr., Lawrence, KS, 66045, United States of America; 2 Department of Chemical and Biomolecular Engineering, Johns Hopkins University, 3400 North Charles St., Baltimore, MD, 21218, United States of America; 3 Department of Molecular Biosciences, University of Kansas, 2030 Becker Dr., Lawrence, KS, 66045, United States of America; University of Michigan, UNITED STATES

## Abstract

Over the past decade, protein-protein interactions have emerged as attractive but challenging targets for therapeutic intervention using small molecules. Due to the relatively flat surfaces that typify protein interaction sites, modern virtual screening tools developed for optimal performance against “traditional” protein targets perform less well when applied instead at protein interaction sites. Previously, we described a docking method specifically catered to the shallow binding modes characteristic of small-molecule inhibitors of protein interaction sites. This method, called DARC (*D*ocking *A*pproach using *R*ay *C*asting), operates by comparing the topography of the protein surface when “viewed” from a vantage point inside the protein against the topography of a bound ligand when “viewed” from the same vantage point. Here, we present five key enhancements to DARC. First, we use multiple vantage points to more accurately determine protein-ligand surface complementarity. Second, we describe a new scheme for rapidly determining optimal weights in the DARC scoring function. Third, we incorporate sampling of ligand conformers “on-the-fly” during docking. Fourth, we move beyond simple shape complementarity and introduce a term in the scoring function to capture electrostatic complementarity. Finally, we adjust the control flow in our GPU implementation of DARC to achieve greater speedup of these calculations. At each step of this study, we evaluate the performance of DARC in a “pose recapitulation” experiment: predicting the binding mode of 25 inhibitors each solved in complex with its distinct target protein (a protein interaction site). Whereas the previous version of DARC docked only one of these inhibitors to within 2 Å RMSD of its position in the crystal structure, the newer version achieves this level of accuracy for 12 of the 25 complexes, corresponding to a statistically significant performance improvement (p < 0.001). Collectively then, we find that the five enhancements described here – which together make up DARC 2.0 – lead to dramatically improved speed and performance relative to the original DARC method.

## Introduction

Protein-protein interactions underlie most biological processes [1–3], and as such many of the individual proteins and networks involved in these interactions are implicated in assorted human diseases [4–7]. Modulating key protein interactions using small molecules can provide exciting opportunities to develop novel therapeutics, leading to extreme interest in this target class for drug discovery [8–12]. Whereas almost all drugs currently in the clinic inhibit one of several “traditional” target classes (G protein-coupled receptors, enzymes, nuclear receptors, transporters, and ion channels) [13, 14], protein-protein interactions now stand among a broad new emerging class of “non-traditional” targets [15, 16].

Unlike enzymes and other traditional drug targets, protein surfaces evolved to bind other proteins typically lack the deep pockets used as small-molecule binding sites [17–19]. Surveys of small-molecule inhibitors of protein interactions have revealed that these compounds tend be larger and more hydrophobic than traditional drug-like molecules, and reside in regions of chemical space that are less represented in commercial libraries [20]. Analysis of crystal structures of small-molecule inhibitors bound at protein interaction sites also reveals that they tend to use shallower binding modes, leading to worse ligand efficiency (binding energy per heavyatom) than their counterparts engaging “traditional” targets [21]. An ancillary pathology of these shallow binding modes is that sterics provide fewer clues for correctly docking candidate inhibitors in virtual screens, and accordingly modern virtual screening tools—tools that have been optimized over many years for their performance against “traditional” targets—do not fare as well when asked to identify compounds active against protein interaction sites [21].

**Fig 1:**
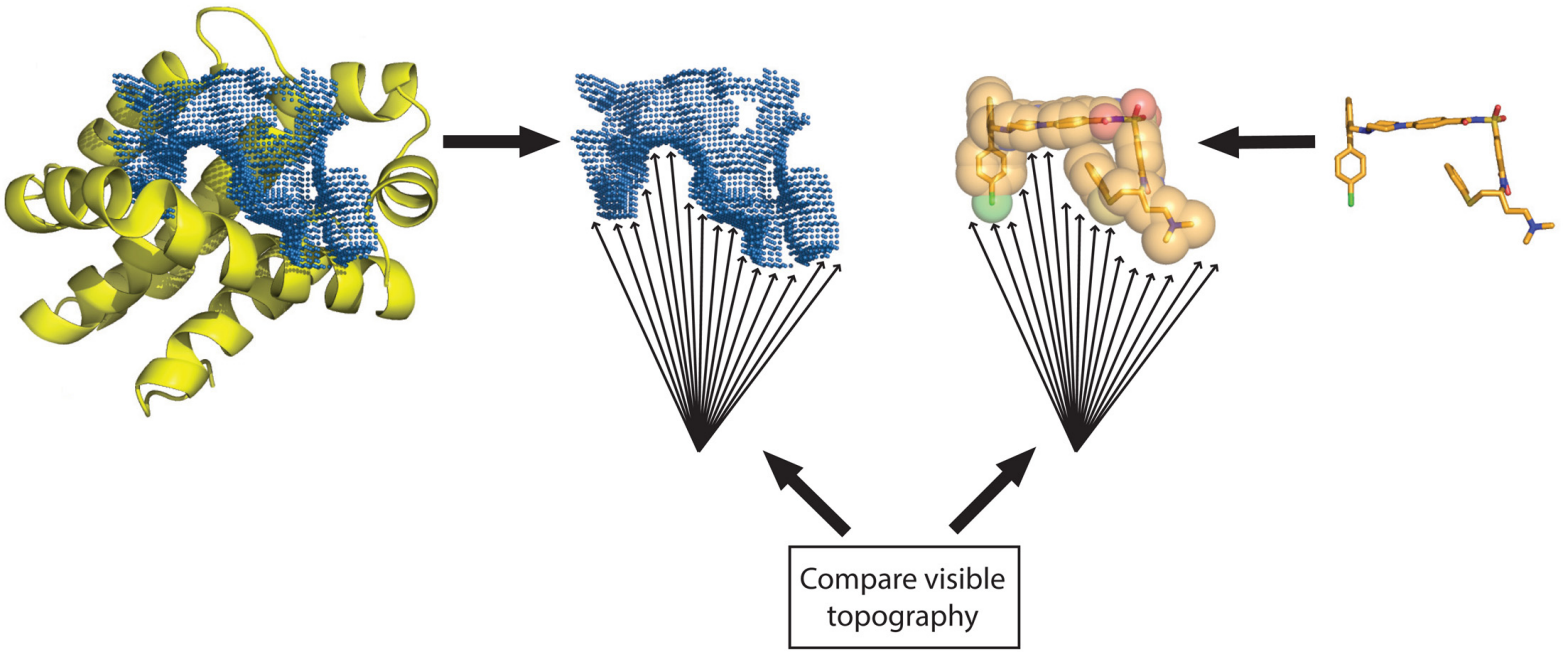
Schematic illustration of the DARC approach. We begin by using a geometry-based method to define the target pocket on the protein surface [24]. Next, we define a series of rays that emanate from a vantage point inside the protein. Collectively, the distance at which these rays reach the protein surface describes the topography of the protein surface in this region, as “seen” from this vantage point. If a bound ligand is complementary to the protein surface, its surface topography will “look” similar to that of the protein surface when viewed from this vantage point; in other words, each ray will intersect the ligand at similar distance as its intersection with the protein surface.

To address this, we recently developed an alternative screening approach called DARC (*D*ocking *A*pproach using *R*ay-*C*asting) [22], a docking method specifically for addressing non-traditional targets such as protein interaction sites. The DARC approach is summarized schematically in Fig 1. We begin by defining the binding pocket around a given “target” residue (or set of “target” residues) on the protein surface, using a pocket-finding algorithm adapted from the LIGSITE program [23] that we have implemented in the Rosetta software suite [24]. To identify a ligand that complements this pocket, we begin by mapping the topography of the pocket by selecting an “origin” point within the protein interior, and casting rays from this origin at each of the pocket points that are in contact with the protein surface. Operationally, this step is equivalent to simply converting each of the pocket points into a spherical coordinate system (ρ,θ,φ) relative to this origin point, where ρ is the distance from the origin point and θ/φ are the polar/azimuthal angles. We additionally include in this step a layer of points outside the pocket, to help define the pocket’s boundaries.

If a ligand docked into this surface pocket is indeed shape-complementary, the surface of this ligand when “viewed” from this vantage point (the origin) should have a very similar topography to that of the pocket. To map the topography of the ligand, we cast a collection of rays from the origin point, with each of the angles (θ,φ) used to map the pocket topography. For each ray, we determine the distance of its first intersection with the ligand (if indeed the ray intersects the ligand). We express the difference in the “observed” surface topographies of the pocket and ligand as follows:

$$Shape\;score\;=\;\sum_{rays}\left\{\begin{array}{c} c1*\left(\rho_{ligand}-\rho_{pocket}\right)\\ c2*\left(\rho_{pocket}-\rho_{ligand}\right)\\ c3\\ c4 \end{array}\;\;\begin{array}{c} if\;\rho_{pocket}<\rho_{ligand}\\ if\;\rho_{ligand}<\rho_{pocket}\\ if\;ray\;does\;not\;intersect\;ligand\\ if\;ray\;does\;not\;intersect\;pocket \end{array}\right\}$$ (1)

Conceptually, each of the four conditions above represents a distinct type of deviation between the two surfaces. In the first case (c1), a ray hits the surface pocket before reaching the ligand: this indicates underpacking in the protein-ligand interface. In the second case (c2), a ray intersects the ligand before reaching the pocket: this indicates that the ligand’s volume overlaps that of the protein, and thus points to a steric clash. In the third case (c3), a ray that intersects the pocket does not intersect the ligand at all: this indicates that the ligand is too small for the pocket. Finally, in the fourth case (c4), a ray that intersects the ligand does not intersect the pocket: this indicates that the ligand extends outside the binding pocket.

Beyond simply evaluating the shape complementary of a protein-ligand complex, this “shape score” can also be used as an objective function for optimization. By adjusting the ligand position and orientation to minimize this score, one can use DARC to rapidly dock a ligand into a surface pocket. In practice, we use a particle swarm optimization (PSO) scheme [25] to minimize the DARC scoring function: much akin to a genetic algorithm, this approach maintains a set of candidate solutions. Each candidate solution corresponds to the translation and rotation relative to a reference ligand position, and thus (indirectly) encodes the bound pose. The position and orientations of the candidate solutions adapt in concert with one another over multiple iterations, and ultimately the “swarm” of solutions ideally converges to an optimal solution (in this case the lowest-scoring pose).

Extending the approach further, by sequentially docking a large number of compounds in this manner one can use DARC to carry out a virtual screen to identify compounds that complement the shape of some surface pocket on a protein of interest. Previously, we found that DARC could be used in virtual screening benchmarks to pick out known small-molecule inhibitors of Bcl-xL and XIAP that were hidden amongst pools of “decoy” compounds. Despite its relatively simple implementation, DARC outperformed other popular virtual screening tools at this task—tools that have been developed in the context of “traditional” target classes, rather than for inhibitors of protein interactions. We then used DARC to carry out a computational screen of 65,000 compounds to identify those that would best complement a pocket on the surface of Mcl-1, an anti-apoptotic member of the Bcl-2 protein family. We tested the top 21 DARC hits in biochemical assays, and found that indeed 10 of these are inhibitors of Mcl-1, with Ki values ranging from 1.2 to 21 μM for the best 4 compounds. Collectively these results validated DARC for virtual screening at protein interaction sites, and demonstrated its usefulness for identifying new inhibitors acting at these sites [22].

We have drawn upon experiences and observations from our early applications of DARC to enhance its performance through the five key ways we describe in this study: ***(1)*** We refined the ray-casting approach, such that rays emanate from multiple origin points to better map the shapes of the surface pocket and the ligand. ***(2)*** We developed a new strategy for parameterizing DARC in a faster and more robust way, thus enabling a broader and more representative set of protein complexes to be included in training. ***(3)*** We introduced a new scheme to efficiently sample small-molecule conformers “on-the-fly” in a single docking trajectory, rather than sequentially consider each conformer in a separate trajectory. ***(4)*** We incorporated electrostatics into the DARC scoring function, allowing simultaneous optimization of both shape- and electrostatic-complementarity. ***(5)*** We identified the computational performance bottleneck in our previous implementation of GPU-DARC [26], and adjusted the control flow by transferring additional calculations onto the GPU to resolve this bottleneck.

As described below, we examine the effect of each of these enhancements in a “pose recapitulation” benchmark. Due to the unfortunate dearth of examples of small-molecule inhibitors bound at a protein interaction sites, the size of our benchmark set is necessarily limited. This, in turn, limits the observed statistical significance of performance improvements stemming from individual enhancements. As will be demonstrated below, however, the *collective* effect of these enhancements results in a notable increase in the number of testcases that are correctly docked to within 2 Å RMSD, with an overall performance improvement that does rise to the level of statistical significance (p < 0.001). Thus, we can conclude that *together*, these individual enhancements lead to dramatic improvements in DARC’s robustness, accuracy, and speed.

## Results

Previously, we assembled a set of unique proteins for which a crystal structure was available in complex with a small-molecule inhibitor of a protein-protein interaction site [21]. At the time, there were 21 such structures available; since then, 4 additional examples have become available. For the studies we describe here, we make use of this new set of 25 non-redundant complexes in which a small-molecule inhibitor is bound at a protein interaction site (S1 Table).

### Enhancement #1: ray-casting using multiple origins

As described above, the topography of the protein surface is mapped from a vantage point inside the protein, using an “origin” point from which rays emanate. The placement of this origin point is critical to ensuring that the resulting topography map gives an accurate and complete view of the surface pocket—in an intuitive sense, it should be centered “behind” the pocket. For an ideal scenario, in which the surface pocket is a purely concave “dimple” on the surface of a near-spherical protein, the protein’s center of mass can serve as a natural choice for the origin’s location. In practice, however, the pocket shapes are never purely concave, and any ruggedness means that some parts of the protein surface cannot be “seen” from a given vantage point. An incomplete description of the protein surface, in turn, limits the ability of DARC to identify truly complementary ligands.

In order to map the protein surface topography more accurately, we therefore modified the ray casting approach such that rays emanate from multiple origins: we expected this strategy would better “illuminate” all regions of the protein surface. To do so, we begin from a single origin centered 30 Å behind the pocket (see Methods). Rotating about a point fixed at the pocket center, we move the origin point by ±45° in each of two orthogonal directions, to generate four new origin points. In other words, if the z-axis connects the first origin to the pocket center, we rotate in first the xz- and then the yz-plane, by ±45° each time, to place these four additional origins.

**Fig 2:**
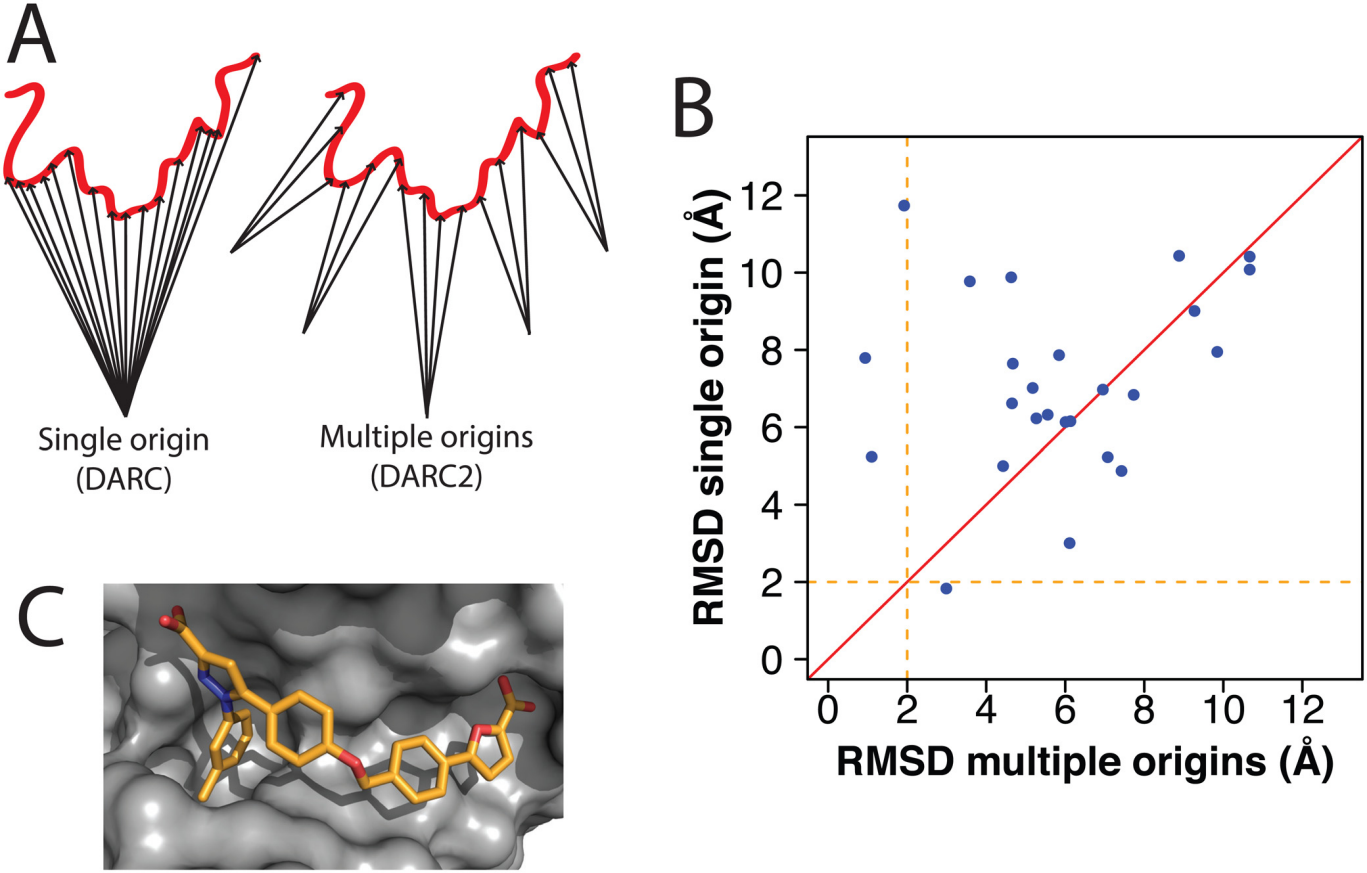
Ray-casting from multiple origins. **(A)** In DARC 2.0, rays emanate from five origin points instead of from a single origin point. This allows more of the protein surface to be “visible” to the rays, and in turn leads to a more complete representation of the surface topography. **(B)** For each protein-ligand complex in our set (S1 Table), we used DARC to dock the ligand back into its cognate receptor. Multiple ligand conformations were considered in the search, while the receptor conformation was held fixed throughout docking. The RMSD of the docked ligand was evaluated relative to its position in the crystal structure of the complex. Each point represents a separate complex; points above the diagonal are those for which the use of multiple origins led to better pose recapitulation. **(C)** A representative example of a complex that was better-predicted using multiple origins (PDB ID 4luz). Part of the binding pocket points directly into the protein (*left side of picture*), and thus directly at the origin (if a single origin is used). The use of multiple origins allows the “walls” of this region of the binding pocket to be included in the surface topography considered for docking.

As before, the topography of the protein surface is mapped by casting rays at each of the “pocket” gridpoints that directly contact the protein. Rather than retain the distances at which rays from all five origins hits a given protein surface point, we only store the distance from the closest origin: each origin is thus “responsible” only for those regions of the protein surface pocket that are closest to it, and for which that origin is therefore likely to have an optimal vantage point (Fig 2A).

Having mapped the protein surface pocket using rays cast from multiple origins, we then evaluate ligand complementarity exactly as described earlier: we simply cast the rays that emanate from each origin towards the ligand, and compare the distances at which these intersect with the ligand to the distances at which they intersected the protein surface (Eq 1). This strategy for using multiple origins does not increase the total number of rays included in DARC’s calculations, but simply distributes the same number of rays among the different origins; accordingly, this new approach does not change the time required for docking using DARC.

To examine the effect of using multiple origins, we used DARC to dock each of the 25 protein-ligand pairs in our test set (S1 Table). In each case we used OMEGA [27–29] to pre-build a set of allowed conformations (“conformers”) for each ligand, and included these in docking; the conformation of the protein was held fixed throughout each simulation. For each of these 25 protein-ligand cognate pairs, we then evaluated the RMSD of the ligand position in the docked complex relative to its position in the corresponding crystal structure.

We also carried out this “pose recapitulation” experiment using the previously described version of DARC [22], which only employed a single origin, and compared the results to those obtained using this new “multiple origins” approach (Fig 2B). We find that the RMSD relative to the crystal structure is better in 16 of the 25 cases (points above the diagonal), suggesting that the use of multiple origins may indeed enable more accurate matching of protein/ligand shape complementarity. Because the 25 complexes in our set represent paired samples (and are not expected to be normally distributed), we employed the (non-parametric and paired) Wilcoxon signed-rank test (see Methods) to compare the results from single versus multiple origins. While this test does identify the difference in performance, it does not quite achieve statistical significance (p < 0.054) due to the relatively small size of the test set (which, in turn, stems from the fact that few examples of crystal structures of small-molecule inhibitors of protein-protein interactions solved in complex with their target proteins are available).

We note that before inclusion of this new feature in DARC, only one complex in our set was docked to within 2 Å RMSD of the crystal structure; using multiple origins, there are now three such cases. Examination of the crystallographic complexes for these improved cases reveals that many share a binding mode in which the ligand (or part of the ligand) faces directly into the protein (Fig 2C, Panel A of S1 Fig). In such cases, rays cast from a single origin would have described only the very bottom of this well; in contrast, the use of multiple origins allow the topography of the walls of this well to also be included, and thus allow the ligand to be more accurately matched to the contours of the protein surface. Nonetheless, there are also 9 (of 25) cases for which performance is slightly diminished. Careful examination of these examples does not reveal any specific characteristics that make these worse; rather, these appear to be comparably docked in terms of shape complementarity (Panel B of S1 Fig), and thus simply arise due to slight shifts in the relative ranking of these mis-docked poses when the origin is altered.

### Enhancement #2: fast and robust weight fitting

There are four parameters (c1/c2/c3/c4) used by DARC when evaluating shape complementarity (Eq 1). Since scaling all four of these by a constant would simply scale the total score, we fix c1 = 1.00 and determine values of the other three parameters accordingly.

In our original parameterization of DARC [22], we used a small training set of seven protein-ligand complexes to optimize these weights. We sought to identify the combination of weights that would optimally allow each of these seven ligands to be docked back into its cognate receptor such that they would match the crystal structures of these complexes. For a given set of weights, then, we assessed performance by using DARC to dock these seven pairs, and used the sum of the resulting seven ligand RMSDs as our objective function. We used simplex optimization to drive our search of parameter space, but each evaluation of this objective function required seven separate calls to Rosetta to carry out the required docking. As a result, carrying out this weight fitting procedure typically required about a week of computation on a modern CPU. Since we originally carried out this parameterization, additional crystal structures of small-molecule inhibitors of protein interactions in complex with their targets have become available; however, our parameterization scheme was already too slow to feasibly add these new examples.

To speed up weight fitting, we adapted our approach such that explicit docking at every step would no longer be required. We also took this opportunity to parameterize DARC not for its ability to simply recapitulate the bound pose of a known ligand, but rather for its ability to optimally distinguish a known ligand from among a pool of “decoy” compounds. The latter represents a virtual screening scenario, such that the resulting weights may exhibit improved performance for this task.

We started by first reformulating DARC’s shape complementarity score as follows:

$$Shape\;score\;=\;c1\;*\;\left(\sum_{\begin{array}{l} rays\;where\\ \rho_{pocket}<\rho_{ligand} \end{array}}\rho_{ligand}-\rho_{pocket}\right)\;+\;c2\;*\;\left(\sum_{\begin{array}{l} rays\;where\\ \rho_{ligand}<\rho_{pocket} \end{array}}\rho_{pocket}-\rho_{ligand}\right)\;+\;c3\;*\;\left(\begin{array}{c} \text{\# rays}\\ \text{that do not}\\ \text{intersect ligand} \end{array}\right)\;+\;c4\;*\;\left(\begin{array}{c} \text{\# rays}\\ \text{that do not}\\ \text{intersect pocket} \end{array}\right)$$ (2)

Relative to our previous formulation (Eq 1), we have simply gathered together the groups of rays that meet each one of the four conditions. This is a natural reformulation of this equation, since the four weights each apply to one of these four conditions.

We again turned to our non-redundant set of 25 complexes in which a small-molecule inhibitor is bound at a protein interaction site (S1 Table); to setup our optimization as a virtual screening problem, we built a “compound library” of 650 diverse decoy ligands, and generated 1000 randomly docked poses for each compound with each protein in this set.

The key to the reformulation of DARC’s shape complementarity score above (Eq 2) is that the result of ray-casting can be separated from the weights: for a given pose we can pre-compute (and store) each of the summations over the rays that meet each of the four conditions. Given some new set of weights, we can then apply these four weights to the four stored numbers and trivially update the score of the pose with these new weights.

Collectively, we generated more than 16 million “decoy poses” (25 proteins x 650 ligands x 1000 randomly docked poses for each protein-ligand combination). Rather than store these decoy poses explicitly, we instead simply stored the four “component energies” (the unweighted terms in Eq 2) for each decoy pose. Similarly, we pre-computed and stored the four “component energies” for each of the 25 native poses.

**Fig 3:**
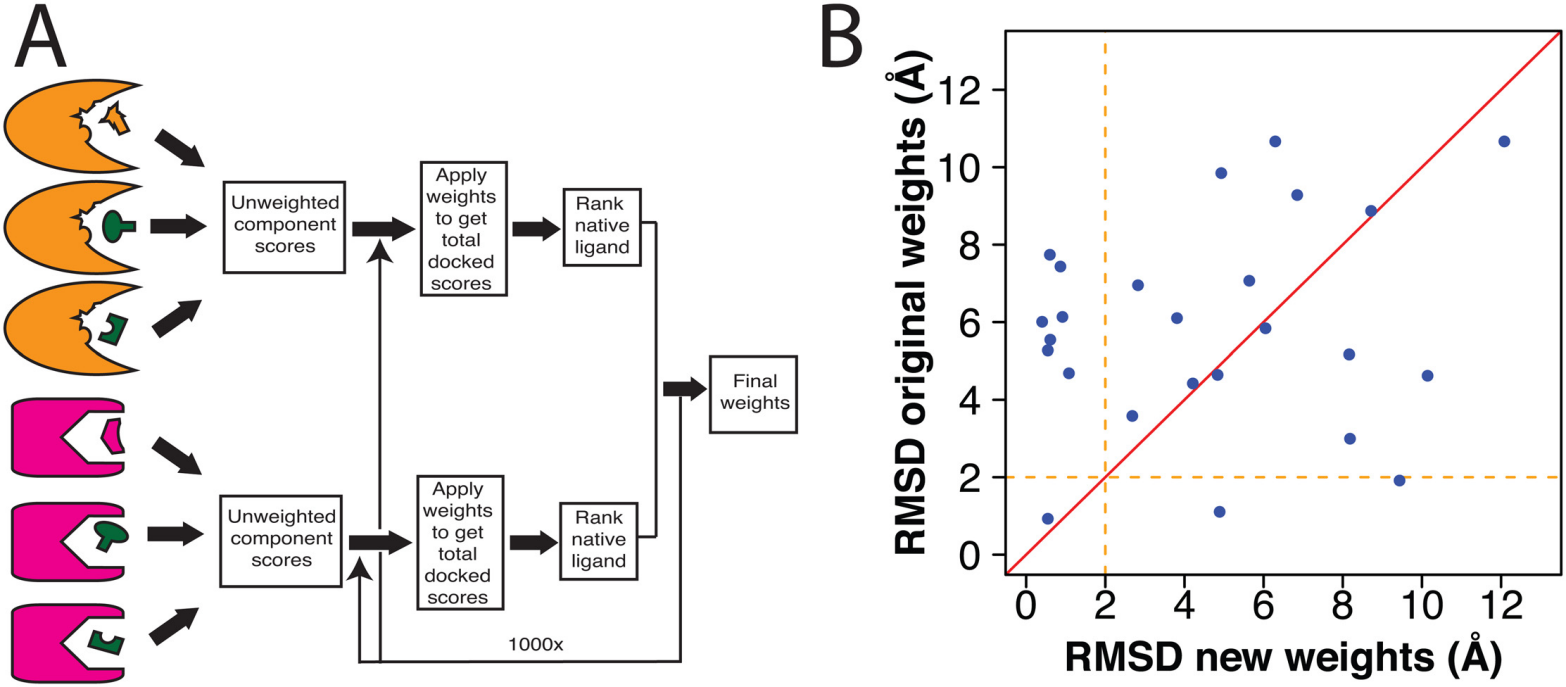
Updated weight-fitting strategy. **(A)** Schematic illustrating the strategy used in DARC 2.0 for determining values of the weights that should be applied to the terms in the DARC scoring function (Eq 1). For each complex used in training (S1 Table), we generate a large set of docked complexes involving either the cognate “native” ligand (*orange/magenta*) or one of many “decoy” ligands (*green*). Using the component energies in these complexes, we identify the set of weights that optimally ranks the native ligands ahead of the “decoys”. **(B)** For each protein-ligand complex in our set, we used DARC to dock the ligand back into its cognate receptor using either the original DARC weights or the newer weights derived though the approach described here. In the latter case, leave-one-out cross-validation was used to ensure the weights were not overfit to the training set. Each point represents a separate complex; points above the diagonal are those for which the new weights led to better pose recapitulation.

Using this data as input, we then carried out weight fitting as shown in Fig 3A. We used simplex optimization (as implemented in the GSL multidimensional minimization library [30]) to search parameter space. At every step, we updated the DARC score for each (decoy and native) complex using the new weights; for each protein target, we then determined the *rank* of the native pose relative to each of the 650,000 decoy poses involving this protein. As the objective function for this minimization, we used the sum of the ranks of the 25 native poses.

This approach to fitting weights required far fewer computational resources than our previous approach; this optimization (using pre-computed component scores) was typically completed in minutes (it took under a minute to run 100 iterations). To objectively examine the performance that could be expected from the weights obtained by this method, we used leave-one-out cross-validation. For each of the 25 proteins in our test set, we developed a unique set of weights trained only on the other 24 proteins; we then used this custom weight set to dock the native ligand of interest back into its cognate receptor, and evaluated the ligand RMSD relative to the crystal structure of this protein-ligand complex. The mean of the custom weight sets used in this experiment are reported in Table 1. The principle change in the updated weight set was a decrease in each term relative to the (fixed) value of c1; collectively, this increases the penalty associated with underpacking at the protein-ligand interface (the contribution scaled by c1).

**Table 1:** DARC weights obtained via different approaches. The first four parameters (c1/c2/c3/c4) refer to those used by DARC to evaluate shape complementarity (Eq 1); the last parameter (c5) is used to scale the electrostatic term, when this term is used (Eq 4). The original DARC weights were obtained by minimizing the collective RMSD for docking a series of seven ligands to their cognate receptors [22]. We also report the weights that arise from our new weight fitting approach (*Enhancement #2*), trained on all 25 proteins in our latest test set. Because this new approach is much faster, we were able to apply leave-one-out cross-validation to this set; here we also report the standard deviation observed among the weights trained on the 24-protein subsets. The magnitudes of the weights are *not* indicative of the relative importance of each term in the scoring function, since the magnitudes of the unscaled contributions vary broadly (e.g. the unscaled electrostatic term is typically much larger than the other terms, so a very small weight is needed to balance its contributions to the total score).

Weight	Original DARC weights	New fitting scheme (no electrostatics)	New fitting scheme (with electrostatics)
c1 (fixed)	1.00	1.00	1.00
c2	3.12	1.7 ± 0.2	1.6 ± 0.2
c3	13.32	4.2 ± 0.7	2.0 ± 0.3
c4	8.13	4.4 ± 1.1	0.8 ± 0.3
c5	N/A	N/A	0.025 ± 0.004

We compared the RMSD of these 25 DARC-docked examples, using our previous DARC weights [22] or using the leave-one-out cross-validated weights from our new approach. We note that results from the earlier weight-fitting approach were not subjected to cross-validation, due to the large computational requirements that would be associated with building numerous “custom” weight sets. We further note that the newer weights are not explicitly trained for docking native complexes, but rather for discrimination in virtual screening tasks. Nonetheless, we find that the newer weight set proves far superior to the original weights (Fig 3B): the ligand RMSD is lower for 17 of the 25 complexes when using the newer weights (points above the diagonal). Further, the newer weights perform exceptionally well in a number of testcases: there are now 8 examples for which the RMSD was less than 2 Å using the new weights, whereas this level of accuracy was only achieved in 3 cases using the older weights. Applying the Wilcoxon signed-rank test to compare the differences in RMSD associated with changing the weights (see Methods) also detects this difference in performance, albeit not quite at a level achieving statistical significance (p < 0.051).

Examination of the crystallographic complexes for these improved cases reveal that indeed the previous weight set may have insufficiently penalized poses that are slightly underpacked (Panel A of S2 Fig). Among the cases for which performance is slightly diminished, on the other hand, there do not appear to be any specific characteristics that make these worse; often, they are simply cases in which the ligand is indeed docked in a shape complementary—but incorrect—pose (Panel B of S2 Fig).

Overall, we attribute the observed improvement to the robustness of the newer weight set, which derives from training on a larger and more broadly representative set of examples; the previous weight set may have been over-fitted to the seven examples in the training set upon which it was based. This is a particularly encouraging outlook in light of the intended use for these weights: they may be far-better suited for virtual screening than the previous weights, since robust tools for this task will require the ability to rapidly and accurately evaluate many diverse ligands (and ideally should prove applicable for diverse protein targets as well).

### Enhancement #3: sampling ligand conformers “on-the-fly”

Efficiently sampling the potential positions, orientations, and conformations of each compound is critical to virtual screening. As the size of “purchasable” chemical space continues to increase [31], and these compounds continue to be a useful for populating *in silico* libraries, the speed of virtual screening is likely to be of paramount importance for the foreseeable future. It is important to note that the speed of modern docking approaches generally scale not only with the size of the screening library, but also with the number of conformations considered for an average ligand in the library.

Modern docking / virtual screening tools address the problem of ligand conformational sampling in different ways. Some programs, such as FRED, pre-generate a collection of low-energy ligand conformations (“conformers”), then sample each of these individually in separate docking trajectories [32]. Other programs, such as AutoDock, generate ligand conformers and evaluate their energy *in situ* during docking [33]. In the DOCK6 program, ligand conformations can either be generated *in situ* during docking with “anchor-and-grow” incremental construction, or alternatively a set of rigid conformers can be pre-generated and screened sequentially [34, 35]. In the original implementation of DARC, no allowance was made for ligand flexibility; alternate ligand conformations were considered by sequentially docking pre-generated conformers, and the best-scoring member of the resulting set was taken to be the final predicted pose [22].

DARC makes use of particle swarm optimization (PSO) [25] to minimize its objective function by varying the ligand’s position and orientation. PSO is a population-based optimization method that mimics swarm intelligence and applies a heuristic approach to find an optimal solution [36]. Others have also used variants of PSO as a fast and efficient optimization method for protein-ligand docking [37–39]. In the case of DARC, we set up the optimization problem such that the displacement and rotation relative to some “reference” position of the ligand are the six degrees of freedom included in the search.

**Fig 4:**
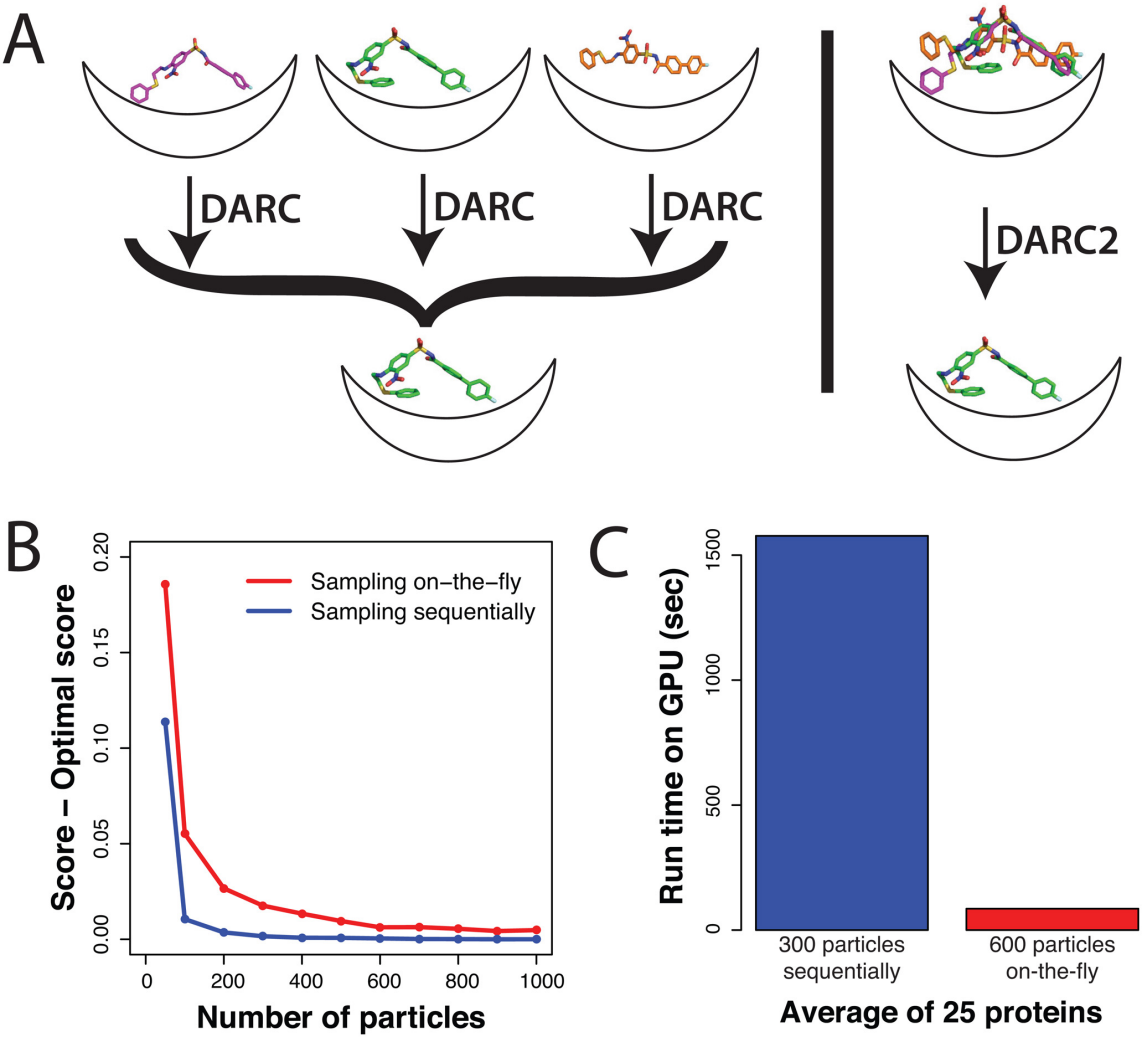
Screening ligand conformers “on-the-fly” during docking. **(A)** Previously, ligand conformations were docked sequentially through separate docking trajectories, and the ligand conformation was ultimately drawn from the best-scoring complex. In DARC 2.0, we instead sample ligand conformers *during* the docking trajectory. **(B)** Due to the extra degree of freedom associated with a single docking trajectory, docking converges more slowly when conformers are sampled “on-the-fly”. Here, convergence is evaluated by the score difference relative to a “gold standard” (best achievable score) for each complex; the results shown are averaged over the 25 complexes in our test set (S1 Table). For each point in this plot, the number of particles in PSO optimization and the number of steps in the docking trajectory were set equal to one another. **(C)** Despite the fact that individual trajectories converge more slowly when conformers are sampled “on-the-fly”, docking is comprised of only a single trajectory. Across our set of 25 complexes, this ultimately makes “on-the-fly” sampling an average of 18-fold faster than sampling conformers sequentially through multiple docking trajectories.

To increase the efficiency of our sampling, we adapted our approach such that the set of (pre-generated) conformers would instead be considered on-the-fly during docking (Fig 4A). We reasoned that not every conformer deserved an equal amount of sampling; by focusing more of our sampling on the top-scoring conformers, the overall time needed for docking a given compound could be reduced. To achieve this, we introduced a seventh degree of freedom in our search: the “conformer index.”

At the start of the simulation, we assigned each of the pre-generated conformers a unique index. During the PSO, seven parameters would be included in the optimization: one to indicate which ligand conformation should be used, and six to transform the atoms of this ligand to the appropriate position and orientation with respect to the protein. This approach is particularly suited to PSO optimization, which simultaneously maintains multiple solutions (“particles”) during a docking trajectory; separate populations that make use of different “promising” conformers can each explore their own local clusters of solution space, whereas conformers that are not used in any productive poses are sampled less frequently. This, in turn, could reduce the overall time required to run the optimization.

Given that the search space for a given trajectory is now much larger (there is an extra degree of freedom), we anticipated that using “on-the-fly” conformer sampling would lead to slower convergence than a trajectory in which only a single conformer was considered. To test this, we first used “sequential” conformer docking with very intensive sampling (1000 particles and 1000 steps) to identify the optimal score that could be obtained when docking the native ligand back into its cognate receptor, for each of the 25 complexes in our set (S1 Table). Next, to assess convergence, we asked how closely the scores for each complex would approach these “gold standard” scores as the amount of sampling was reduced by simultaneously lowering the number of particles and steps in the search.

As expected, we indeed find that convergence to near-optimal solutions occurs more slowly with “on-the-fly” sampling instead of sequential sampling (Fig 4B). Whereas the optimal solutions are obtained using only 300 particles / 300 steps of sequential sampling, 600 particles / 600 steps were required for convergence when using sampling “on-the-fly”. Despite the need for more sampling *per trajectory*, however, the advantage of on-the-fly sampling lies in the fact that *only a single trajectory is needed*. Since we use an average of 163 conformers for the ligands in our test set (S1 Table), and sequential sampling requires that a separate trajectory be carried out for each conformer, the average runtime for sequential sampling is much longer (Fig 4C). Comparing the runtime required for equivalent sampling (300 particles / 300 steps of sequential sampling versus 600 particles / 600 steps of on-the-fly sampling), we find that on average an 18-fold speedup is achieved when on-the-fly conformer sampling is used.

### Enhancement #4: inclusion of electrostatic complementarity

Complementarity between a ligand and its binding pocket on the protein surface is the guiding principle in protein-ligand docking, and the success of DARC to date is based on this fundamental principle. In its original inception, DARC was purely based on optimizing and identifying shape complementarity between the surface of the ligand and the surface of the protein [22]. In addition to shape complementarity, however, the chemical complementarity of the interacting surfaces is clearly essential for protein-ligand recognition. In addition to the well-established electrostatic complementarity between evolved protein-protein binding partners [40, 41], it has more recently been recognized that small-molecule inhibitors of protein-protein interactions sometimes (inadvertently) mimic the electrostatic patterning of the natural binding partner, in order to optimally complement the charge distribution presented by the surface of the target protein [42].

Of course, other docking methods recognize the importance of electrostatic complementarity, and include its contribution through various approaches. Since most virtual screening tools do not incorporate receptor flexibility during docking, typical modern approaches pre-generate an “electrostatic grid map”, and use this to calculate the electrostatic interaction energy given the position of the ligand. Broadly speaking, this is strategy utilized in both AutoDock4 (through AutoGrid) [33, 43] and the DOCK suite [34, 44].

While DARC was originally predicated on matching the surface shapes of the ligand and the protein surface, we quickly noted (by inspection of mis-docked structures) that a number of ligands exhibited pseudo-symmetry when examined purely on the basis of their shapes. In other words, docking without consideration of chemical complementarity very quickly highlighted the limitations of docking on the basis of shape complementarity alone.

**Fig 5:**
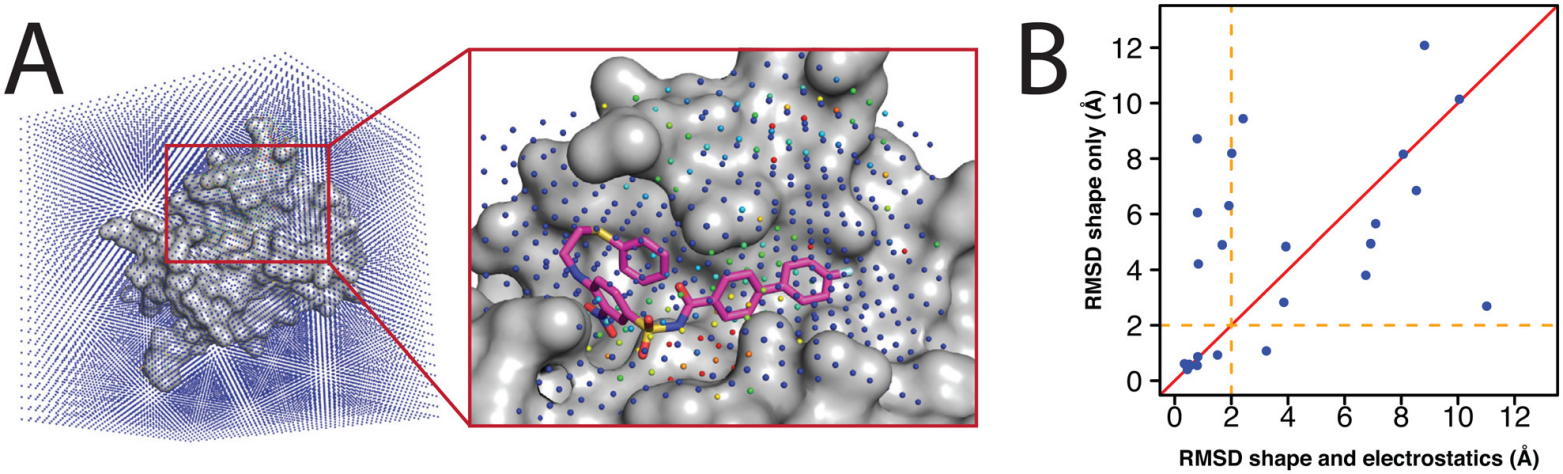
Incorporation of electrostatic complementarity into DARC 2.0. **(A)** The electrostatic potential is evaluated at a series of grid points over the whole protein using the finite difference Poisson-Boltzmann solver included in OpenEye’s ZAP toolkit [45]. We use trilinear interpolation of the closest gridpoints to determine the electrostatic potential at points corresponding to locations of ligand atoms, and then use the ligand partial charges to compute the electrostatic interaction energy (Eq 3). **(B)** For each protein-ligand complex in our set (S1 Table), we used DARC to dock the ligand back into its cognate receptor either with or without including the electrostatic complementarity term. In both cases, leave-one-out cross-validation was used to ensure the weights were not overfit to the training set. Each point represents a separate complex; points above the diagonal are those for which inclusion of electrostatics led to better pose recapitulation.

To address this, we built into DARC the ability to capture electrostatic complementarity using the most common approach employed by other modern docking tools. Given the (fixed) receptor conformation, we solve the Poisson-Boltzmann equation to calculate the electrostatic potential at a series of gridpoints that span the surface pocket of interest (Fig 5A). For convenience and speed, here we used the finite difference Poisson-Boltzmann solver included in OpenEye’s ZAP toolkit [45] for this task (see Methods).

Given the electrostatic potential, we evaluate the electrostatic complementarity by summing over atomic partial charges in the traditional manner as follows:

$$Electrostatics\;score\;=\;\;\left(\sum_{\begin{array}{l} ligand\\ atoms \end{array}}qi\phi i\right)$$ (3)

To determine the electrostatic potential (*ϕ*_*i*_) at the location of a given ligand atom (*i*), we use trilinear interpolation of the closest gridpoints that encapsulate the center of the atom. Atomic partial charges (*q*_*i*_) for the ligand were determined using the “molcharge” program from OpenEye (see Methods). To ensure the ligand remained within the bounds of the protein surface pocket, we set the electrostatic potential to zero in the protein interior, and applied an unfavorable value of the electrostatic potential outside the defined binding site (+100 DARC units • C^-1^).

We then used the strategy described above (*Enhancement #2*) to develop a new set of weights for DARC, this time including electrostatic complementarity as follows:

$$DARC\;score\;=\;c1\;*\;\left(\sum_{\begin{array}{l} rays\;where\\ \rho_{pocket}<\rho_{ligand} \end{array}}\rho_{ligand}-\rho_{pocket}\right)\;+\;c2\;*\;\left(\sum_{\begin{array}{l} rays\;where\\ \rho_{ligand}<\rho_{pocket} \end{array}}\rho_{pocket}-\rho_{ligand}\right)\;+\;c3\;*\;\left(\begin{array}{c} \text{\# rays}\\ \text{that do not}\\ \text{intersect ligand} \end{array}\right)\;+\;c4\;*\;\left(\begin{array}{c} \text{\# rays}\\ \text{that do not}\\ \text{intersect pocket} \end{array}\right)\;+\;c5\;*\;\left(\sum_{\begin{array}{l} ligand\\ atoms \end{array}}qi\phi i\right)$$ (4)

Critically, we note that the same reformulation that enabled the weight-fitting strategy described earlier (decoupling the energetic contributions from their weights) applies equally well here; this allowed us to use the same approach to derive a new set of weights that includes this electrostatic term. For the results presented below, we again used the same leave-one-out cross-validation described earlier.

As we had done after each of the previous enhancements, we returned to the 25 complexes in our test set (S1 Table), and used this latest iteration of DARC to dock multiple conformations of each ligand against its cognate protein partner (Fig 5B). Previously, on the basis of shape alone, we found that the RMSD of the docked ligand relative to the crystal structure was less than 2 Å in 8 cases. Upon inclusion of electrostatics, 7 of these remain “correctly docked” while the RMSD in one case increases above 2 Å. Of the cases that were *not* previously docked to within 2 Å RMSD, however, five new complexes were now “correctly docked” upon inclusion of this electrostatics term (for a total of 12 such cases). Examination of such cases showed that these were typically ligands bound to flat regions of the protein surface; while shape alone was insufficient to correctly dock the ligand, inclusion of electrostatics enabled the native pose to be identified (Panel A of S3 Fig). Of the cases for which performance was slightly diminished, we find relatively non-polar protein surfaces with nearly symmetric binding pockets; in this case, formation of an incorrect hydrogen bond led to selection of the wrong pose (Panel B of S3 Fig).

Applying the Wilcoxon signed-rank test to compare the differences in RMSD associated with inclusion of electrostatics (see Methods), the improvement is detected but again does not reach statistical significance (p < 0.156) due to the modest size of our test set.

### Enhancement #5: improved implementation for GPU computing

The fact that graphics processing units (GPUs) were originally designed to process multithreaded 3D graphics through ray-tracing makes them extremely well-suited for the ray-casting that underlies DARC. Previously, we adapted DARC such that the ray-casting step would be carried out on the GPU; meanwhile, the central processing unit (CPU) would be responsible for updating the ligand coordinates and repeatedly passing these to the GPU. This GPU implementation proved extremely useful, because it led to a speedup of about 27-fold in typical-use cases, as compared to the time required to carry out the analogous calculations using the CPU alone [26].

Upon more recent examination of the speedup observed when carrying out various calculations in DARC, we found that the size of the ligand and the number of particles both contributed to the bottleneck in the speedup that could be achieved. As noted earlier, DARC uses particle swarm minimization to optimize the ligand’s displacement and rotation (and now the “conformer index” as well, for on-the-fly sampling) relative to a saved “reference” position. While the ray-casting step was taking place on the GPU, applying the transformation to translate and rotate the ligand to its new coordinates was carried out on the CPU, and was required for every particle (at every step of the docking trajectory). Our observations of the scaling with respect to ligand size and number of particles led us to hypothesize that the performance bottleneck in the GPU-enabled calculation was either due to the time required for the CPU to apply the appropriate transformation to every ligand atom of every particle, or because of the amount of data transferred from the CPU to the GPU.

**Fig 6:**
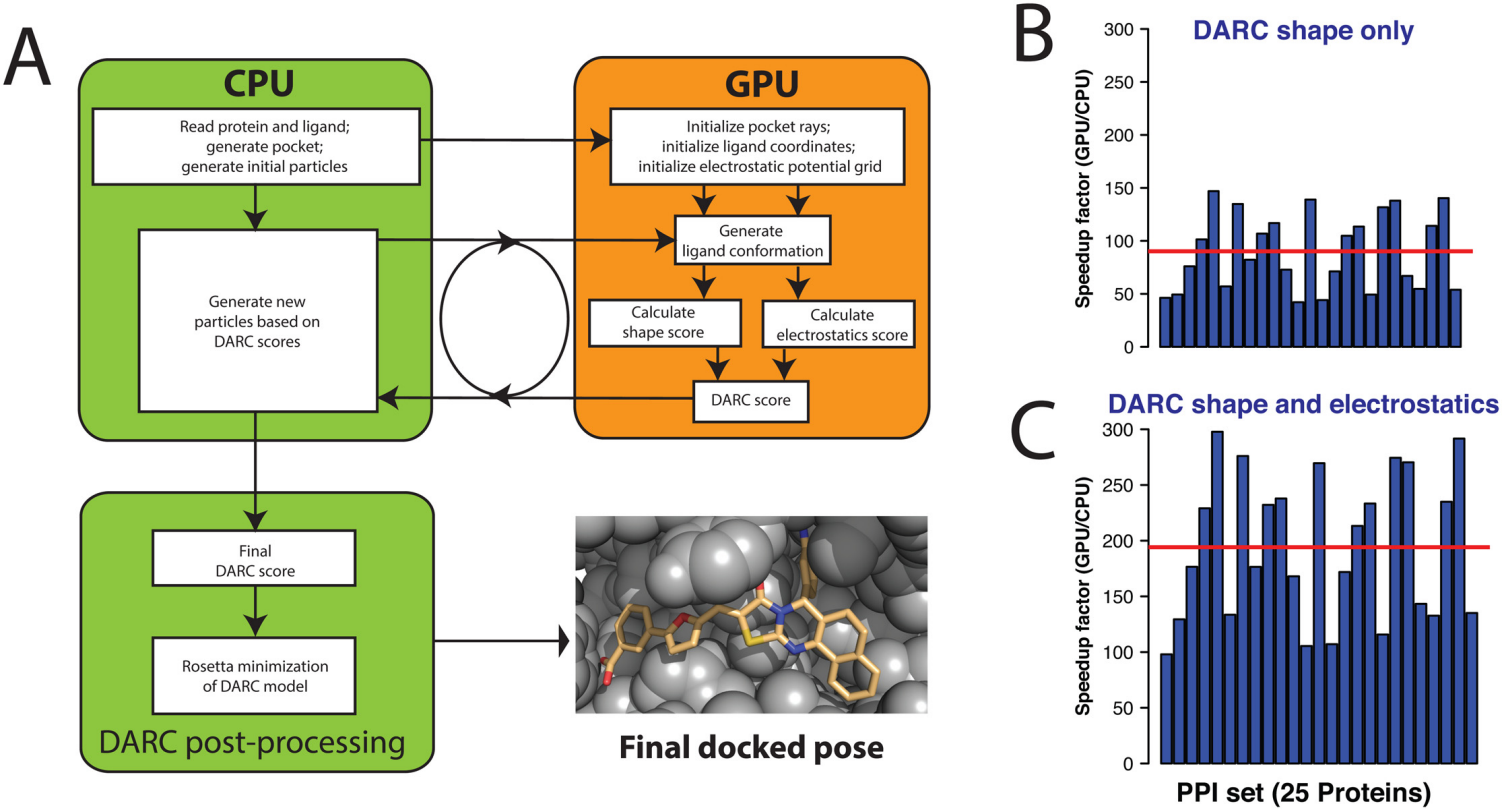
Updated GPU control flow. **(A)** Schematic illustration of CPU-GPU control flow in DARC 2.0. Previously, the ligand conformation was generated on the CPU and passed to the GPU; now, the conformer index / displacement / rotation (relative to a “reference” position) is instead passed, and the GPU is responsible for applying this transformation to the ligand’s atomic coordinates. The new electrostatic complementarity term is also computed entirely on the GPU. **(B)** For each protein-ligand complex in our set (S1 Table), we timed DARC when docking the ligand back into its cognate receptor, using either GPU+CPU or CPU alone. We find an average speedup of 90-fold when using the GPU (red line), an improvement over the 27-fold speedup we achieved in our original GPU implementation of DARC [26]. **(C)** The GPU led to an even greater speedup over the analogous calculation on the CPU when electrostatic complementarity was included in both calculations (190-fold speedup).

To address this bottleneck, we devised a new scheme for splitting control flow between the CPU and the GPU (Fig 6A). During setup, our new approach stores the “reference” position of each ligand conformer on the GPU. At each step of a docking trajectory, we previously passed from the CPU to the GPU a message obtained by “unpacking” the information in each particle (the coordinates of each ligand atom for that particle); now, we instead pass only the seven numbers stored in each particle: the conformer index (1 number), the displacement that must be applied to the ligand’s reference conformation (3 numbers), and the rotation that must be applied to the ligand’s reference conformation (3 numbers). In addition to reducing the amount of information transferred, this also allows the transformations of the ligand coordinates to be carried out on the GPU in a highly parallel fashion (instead of carrying out this step sequentially on the CPU).

To evaluate the speedup achieved by this new strategy we determined the time needed for docking each of the 25 complexes in our test set (S1 Table), either using a CPU alone or using GPU-enabled DARC (Fig 6B). Unsurprisingly, we find that the ratio of the runtimes (the “speedup factor”) differs for the complexes in our set: the size and shapes of the pockets differ (causing the number of rays to differ), and the ligand sizes differ. Nonetheless, on average we observe a 90-fold speedup when running on the GPU—about three times faster than our original GPU implementation. This result confirms our identification of the previous performance bottleneck, which has been successfully overcome through this new CPU/GPU control scheme.

Our scheme also proved naturally amenable for using the GPU to calculate the electrostatic part of the DARC score as well (Fig 6A): at setup, we simply store the electrostatic potential grid on the GPU, and use the atomic positions of the ligand to compute the electrostatic score as described earlier (Eq 3). This part of the calculation also benefits tremendously from GPU parallelization: when electrostatic complementarity is included in the calculation, the average speedup of GPU-enabled DARC (relative to the analogous calculation on CPU alone) reaches 190-fold (Fig 6C).

## Discussion

**Fig 7:**
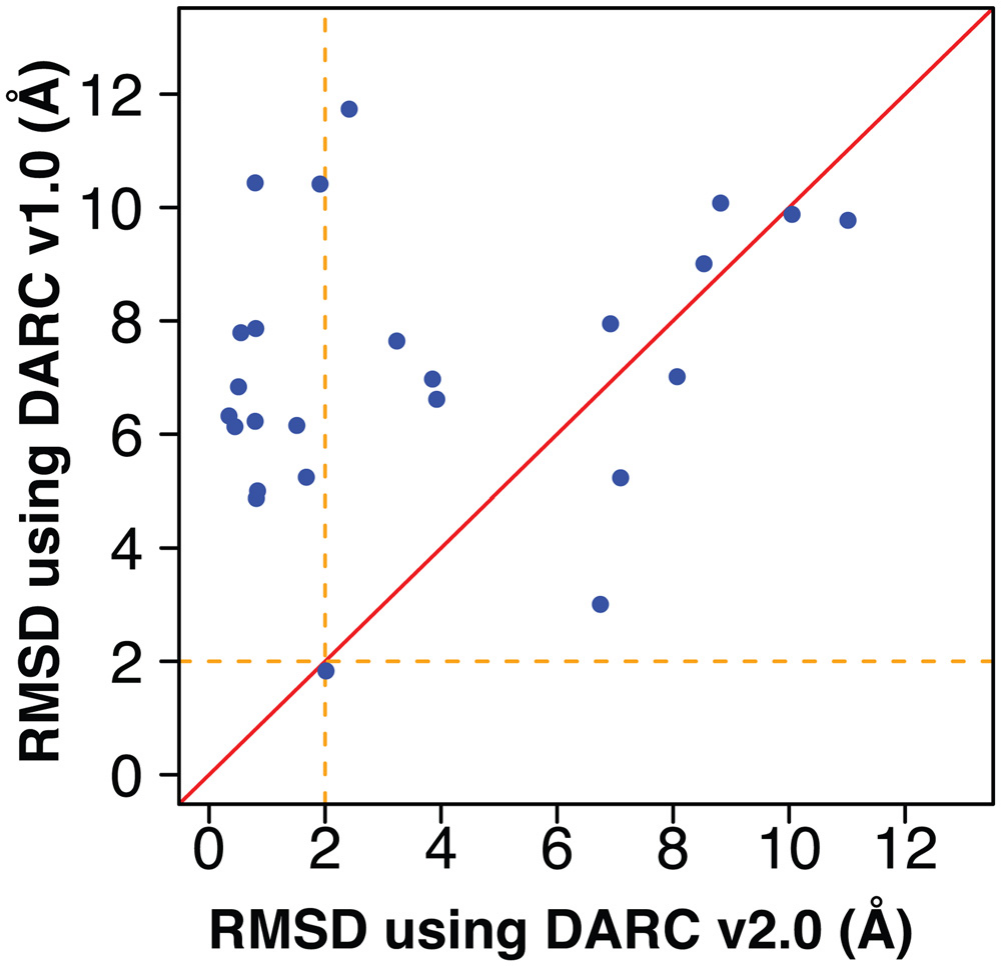
Summary of the collective effect of enhancements to DARC described here. For each protein-ligand complex in our set (S1 Table), we used DARC to dock the ligand back into its cognate receptor. Here we compare performance of DARC before the enhancements described in this work (“DARC 1.0”), to its current performance (“DARC 2.0”). Each point represents a separate complex; points above the diagonal are those for which the use of multiple origins led to better pose recapitulation. Previously, only one ligand (of 25) was docked to within 2 Å RMSD of its position in the crystal structure; in contrast, “DARC 2.0” now achieves this level of accuracy in 12 cases.

Here, we present a number of enhancements to the robustness, speed and accuracy of DARC; each enhancement builds upon the previous one. These include introduction of multiple origins from which rays can emanate, a new scheme for rapidly determining optimal weights in the scoring function, the ability to rapidly screen conformers “on-the-fly” during docking, inclusion of electrostatic complementarity in the scoring function, and improved control flow for GPU computing. As a result of the linear narrative by which we have describe these enhancements, however, the overall improvement from this collection of improvements is less apparent. In Fig 7, we therefore re-plot the results of our docking experiment such that we compare the results from this latest, fully-enhanced version of DARC—which we call “DARC 2.0”–against the iteration of DARC described in our previous work [22] (“DARC 1.0”) that marked the starting point for the current study. Whereas our starting version of DARC docked only *one* of the ligands in our test set to within 2 Å RMSD of its position in the crystal structure, “DARC 2.0” achieves this level of accuracy for *12* of the 25 complexes. The dramatic improvement in the RMSD of these docked complexes is reflected through the Wilcoxon signed-rank test, which confirms a statistically significant performance improvement from these collective enhancements (p < 0.001).

Even with these enhancements, the performance of DARC on this benchmark is worse than that reported for benchmarks comprised of “traditional” drug targets such as enzymes and hormone receptors: pose prediction experiments find that several methods can successfully reproduce the bound pose for these targets to within 2 Å RMSD upwards of 70% of the time [46], though the results do vary based on the nature of the targets themselves [47]. The difficulty associated with our benchmark set (small molecules that inhibit protein-protein interactions) was not unexpected, based on our previous observation that other docking methods also struggle with this target class: in a virtual screening experiment, we found the native ligand ranked in the top 2% for 80% of the targets in a “traditional” set, compared with only 50% of the targets in a set of protein interaction inhibitors [21]. It is thus unsurprising that the challenges associated with docking to the surfaces typical of protein interaction sites are reflected in the overall success rate of this experiment.

We also note that our benchmark included the crystallographic (protein-bound) ligand conformers among those sampled: in a “true” application, such a structure would not be available. In this experiment, we included the “correct” ligand conformer in order to avoid apparently negative results for cases in which no ligand conformer at all was available within 2 Å RMSD of the crystallographic ligand. As shown in S1 Table, in fact there were three cases in which *none* of the (non-crystallographic) ligands generated by OMEGA were within 2 Å RMSD of the crystallographic ligand. In such cases, even a perfectly docked conformer could not have achieved our goal of docking to within 2 Å RMSD of the crystallographic ligand. In S4 Fig, we show the results of docking using only the OMEGA-generated conformers: again our starting version of DARC docked only one of the ligands in our test set to within 2 Å RMSD of its position in the crystal structure, and while the overall improvement is still evident, “DARC 2.0” now achieves this level of accuracy in fewer cases (7 of 25 complexes). In retrospect, this highlights the challenges facing modern tools that generate reasonable (likely bio-active) conformations of small molecules; further advances in pose recapitulation by docking tools may require corresponding advances in generating “near-correct” conformers.

Nonetheless, the ability to achieve such a dramatic collective improvement in DARC 2.0 relative to DARC 1.0 is striking in part due to the success of DARC 1.0 for virtual screening. As noted earlier, our initial deployment of DARC in a screen against Mcl-1 allowed us to identify 4 new inhibitors with Ki values ranging from 1.2 to 21 μM [22]; given that we tested 21 compounds suggested by DARC, this corresponded to a success rate of 19% at this potency cutoff. In retrospect, the unimpressive performance of DARC for pose recapitulation did not foretell poor performance in this seemingly more challenging arena, because in fact virtual screening is—in many ways—an easier task.

In a virtual screening experiment, two types of errors can limit performance: false negatives (compounds that do not receive a high ranking, though they are in fact active) and false positives (compounds predicted to be active that are not actually active). In practice, as long as *some* hits are identified then a screening campaign is considered successful: missing out on additional active compounds in the library does not detract from this success. In other words, provided that the number of “true” hits in a library is not exceedingly small, false positives limit the perceived performance of virtual screening much more than false negatives. This is deceptive in some ways, however, since these additional hit compounds that were (incorrectly) excluded may have superior potency than the compounds that were ultimately prioritized for further characterization (i.e. in “wetlab” assays).

In a pose recapitulation benchmark, such as the one used in this study, the ligand to be used for each testcase is pre-determined, and there is a single “right answer” (i.e. the bound pose from the crystal structure). This is a far more stringent test than a virtual screen; when screening, failing to correctly dock an individual (active) compound from the library would simply lead to exclusion of this compound from among the hits (a false negative), and would go unnoticed.

In retrospect, DARC 1.0 exhibited impressive performance for virtual screening because a number of active compounds were identified—but many other compounds more potent than those we chose to characterize may have been present in our library. Because of the improved performance demonstrated by DARC 2.0 in pose recapitulation, we anticipate fewer false negatives in screening applications—leading, in turn, to improved potency of initial screening hits from DARC 2.0 relative to DARC 1.0.

## Methods

### Implementation in Rosetta

DARC is implemented as part of the Rosetta macromolecular modeling suite [48]. Rosetta is freely available for academic use (www.rosettacommons.org), with the new features described here included in official releases 2015.05 and beyond. The Protocol Capture accompanying this manuscript (S1 Dataset) contains all the commands required for running DARC, including sample input and output files. All results reported here were generated using *git revision 011e012* of the master source code.

### Running DARC with Rosetta (no electrostatics)

Running DARC within Rosetta is a two-step process: first generating the ray file, and second docking with DARC.

In the first step we generate the protein surface pocket and map the shape of the pocket shell (points in direct contact with the protein) to a spherical coordinate file; we call this a “ray-file”. To generate this ray-file we need to input the protein (in PDB format), and specify one or more target residue(s). The command to run DARC is as follows:

Rosetta/main/source/bin/make_ray_files.macosclangrelease-protein 4ERF.pdb

-central_relax_pdb_num 54,99

To use multiple origins, we use:

Rosetta/main/source/bin/make_ray_files.macosclangrelease-protein 4ERF.pdb

-central_relax_pdb_num 54,99 –multiple_origin

In the second step, we are actually running the docking calculations using the pre-generated ray-file. Here we give the input ligand(s) for screening against the ray-file, as follows:

Rosetta/main/source/bin/DARC.macosclangrelease-protein 4ERF.pdb-ligand 0R3_0001.pdb

-extra_res_fa 0R3.params-ray_file eggshell_rosetta_4ERF_54,99.txt

To search conformers on-the-fly:

Rosetta/main/source/bin/DARC.macosclangrelease-protein 4ERF.pdb-ligand 0R3_0001.pdb

-extra_res_fa 0R3.params-ray_file eggshell_rosetta_4ERF_54,99.txt
–search_conformers true

Rather than center the pocket grid at the target residue(s), we can instead center it using a bound ligand (primarily for benchmarking purposes):

Rosetta/main/source/bin/make_ray_files.macosclangrelease-protein 4ERF.pdb

-central_relax_pdb_num 54,99 -bound_ligand 0R3_0001.pdb
-extra_res_fa 0R3.params –lig_grid

The output of the DARC run is a docked model of the protein-ligand complex; in this case it would be named “DARC_4ERF_0R3.pdb”

### Fullatom minimization in Rosetta

Fullatom minimization of the DARC models can either be carried out separately in Rosetta, or immediately after completion of the DARC. To minimize the DARC models immediately after docking we add the flag “-minimize_output_complex” as follows:

Rosetta/main/source/bin/DARC.macosclangrelease-protein 4ERF.pdb-ligand 0R3_0001.pdb

-extra_res_fa 0R3.params-ray_file eggshell_rosetta_4ERF_54,99.txt
–minimize_output_complex

This gives an additional output file named “mini_4ERF_0R3.pdb”.

Other optional flags to use when running DARC include:

-origin_cutoff 9-atom_radius_scale 0.9 -num_particles 100 -num_runs 100
-missing_point_weight 13.3-steric_weight 3.12-extra_point_weight 8.13
–esp_weight 0.03 –use_connolly_surface

### Multiple origin points

Whether using a single origin or multiple origins, we begin by placing the first origin point (O_1_) at a distance 30 Å from the center of the pocket, and at a location centered “behind” the pocket. When using a single origin, we noted that the location of the origin is key for suitably defining the topography of the protein surface. The protein center of mass can work well for globular proteins, but can be “off-center” for many proteins that are not nearly-spherical. Below we describe several ways to define O_1_: their applicability depends in part on the geometry of the pocket itself. We note, however, that the use of multiple origins provides more robust results with respect to the location of O_1_.

As a first step, the user can choose whether O_1_ should simply be placed in the direction of the protein’s center of mass (this is default). If so, we place O_1_ 30 Å away from the center of the pocket (P) along the P→Q direction, where Q is the center of the protein. If not, we offer three distinct methods to set O_1_: ***(1)*** We make use of the fact that pockets at protein-interaction sites are broad and flat, and thus we find the plane that best fits the pocket points (by minimizing the least-squares distance of points to the plane). We then place O_1_ along the normal to the plane passing through P, so that distance P–O_1_ = 30 Å, yielding two solutions (one “above” the plane of the pocket and one “below” the plane of the pocket). We then select the solution for which the O_1_–Q distance is less (i.e. the rays will emanate from within the protein rather than from far above the pocket). ***(2)*** Alternatively, for pockets that are deeper and narrower, we define a series of vectors **s**_i_, each of which defines the distance and direction from the *i*_th_ surface point to P. We then carry out a vector summation of all these vectors **s**_i_, such that we determine the direction of the pocket which most faces away from solvent. We place O_1_ in this direction at a distance of 30 Å. ***(3)*** Finally, we offer the user fine control over the location of the origin by placing O_1_ 30 Å away from P along the P→R direction, where R is a user-specified residue.

Once O_1_ is defined, four more origin points (O_2_–O_5_) are then defined. We add O_2_–O_5_ as follows: O_2_ and O_3_ are obtained by rotating O_1_ by ±45° around vector **w** = **u**×**v**, where **u** is the vector from P to O_1_, **v** is the vector from P to a randomly chosen point, and × denotes vector product; O_4_ and O_5_ are obtained by rotating O_1_ by ±45° around vector **z** = **u**×**w**.

### Electrostatic potential grid

To prepare the protein, we begin by using Rosetta to fill in any missing atomic coordinates and add hydrogen atoms. We then use OpenEye’s “molcharge” program to add amber99 partial charges to each atom.

To generate the electrostatic potential grid, we use OpenEye’s ZAP toolkit [45] (a finite difference Poisson-Boltzmann solver). We use 0.5 Å grid spacing, with 1.0 and 80.0 for the inner and outer dielectrics, and 2 Å distance as buffer between the molecule and the edge of the grid. Once we obtain the electrostatic potential grid that encompasses the whole protein, we extract from this a smaller grid that matches the dimensions of the “pocket grid” used for ray-casting (this also matches the bounds of the search space during the docking runs). To avoid extreme values that occur at certain grid points (i.e. very close to a charged atom) during docking, we set the maximum/minimum possible value of the electrostatic potential at each point to ±10 kT/e.

### Running DARC with Rosetta (including electrostatics)

To include electrostatics, we first resize the electrostatic potential grid (generated as described above) to match the size of the interface pocket grid. This step can be carried out while generating the ray file:

Rosetta/main/source/bin/make_ray_files.macosclangrelease-protein 4ERF.pdb

-central_relax_pdb_num 54,99-bound_ligand 0R3_0001.pdb
-add_electrostatics –espGrid_file 4ERF.agd-extra_res_fa 0R3.params

The output from this command will be a ray-file named “eggshell_4ERF_54,99.txt” and an electrostatic potential grid file named “DARC_4ERF.agd” which we will use as input for running docking using DARC.

Then we call DARC for running the docking calculations using the pre-generated ray-file and corresponding electrostatic potential grid as follows:

Rosetta/main/source/bin/DARC.macosclangrelease-protein 4ERF.pdb-ligand 0R3_0001.pdb

-extra_res_fa 0R3.params-ray_file eggshell_rosetta_4ERF_54,99.txt

To include electrostatics score:

Rosetta/main/source/bin/DARC.macosclangrelease-protein 4ERF.pdb-ligand 0R3_0001.pdb

-extra_res_fa 0R3.params-ray_file eggshell_rosetta_4ERF_54,99.txt
–add_electrostatics -espGrid_file DARC_4ERF.agd

### Generating conformers

For each ligand in our test set, we used the OMEGA software [27–29] to generate up to 300 conformers, using default parameters. The number of conformers used for each ligand in our study is reported in S1 Table (these depend on the number of rotatable bonds and the ligand’s geometry).

### Statistical analysis

The statistical significance of the comparisons presented here was evaluated using Wilcoxon signed-rank test, as implemented in the R statistical computing environment [49].

## Supporting Information

S1 Dataset Protocol Capture. The protocol capture contains all the example input and output files, and representative command-lines and flags required for running DARC.
(GZ) Click here for additional data file.

S1 Fig Selected examples responding to ray-casting from multiple origins. In all cases, the crystallographic ligand is shown in *green*, while the docked model is shown in *cyan*. **(A)** In this example (4LUZ, also shown in Fig 2C), the crystallographic ligand includes a ring facing directly into the protein (at the left side, in this perspective). The use of multiple origins captures the “walls” of this well, leading to a much-improved pose (RMSD goes from 11.7 Å to 1.9 Å). **(B)** Among examples for which performance was slightly deteriorated when using multiple origins (e.g. 2KP8, RMSD goes from 1.8 Å to 3.0 Å), the pockets were typically relatively flat and featureless; both docked poses appear to have equivalent shape complementarity, and the difference is presumably due simply to slight shifts in the relative ranking of these mis-docked poses when the origin is altered.
(TIFF)

S2 Fig Selected examples responding to the updated weights. In all cases, the crystallographic ligand is shown in *green*, while the docked model is shown in *cyan*. **(A)** In this example (2YEL), the previous weight set may have insufficiently penalized underpacking at the protein-ligand interface. The new weights increase this penalty, leading to a much-improved pose (RMSD goes from 6.0 Å to 0.4 Å). **(B)** Among examples for which performance was slightly deteriorated when using the newer weight set (e.g. 1ALW, RMSD goes from 1.1 Å to 4.9 Å), the based for the diminished performance is not clear; both docked poses appear to have equivalent shape complementarity, and the difference is presumably due simply to slight shifts in the relative ranking of these mis-docked poses.
(TIFF)

S3 Fig Selected examples responding to inclusion of electrostatic complementarity. In all cases, the crystallographic ligand is shown in *green*, while the docked model is shown in *cyan*. **(A)** In this example (3IN7, RMSD goes from 8.7 Å to 0.8 Å), the crystallographic ligand binds to a flat region of the protein surface. Using shape alone, the correct pose is not clear; upon inclusion of the electrostatics term, the correct pose can be identified based on interactions around the (charged) phosphate group (at the left side of the native pose, in this perspective). **(B)** Among examples for which performance was slightly deteriorated when including electrostatics (e.g. 1YSI, RMSD goes from 2.7 Å to 11.0 Å), we find relatively non-polar protein surfaces with nearly symmetric binding pockets. While the sulfonamide group makes favorable electrostatic interactions in the native pose, the incorrect pose selected upon inclusion of electrostatics includes alternate (equally favorable) electrostatic interactions involving this group.
(TIFF)

S4 Fig Summary of the collective effect of enhancements to DARC, when the crystallographic conformer is *not* included in ligand sampling. As in Fig 7, this plot compares the performance of DARC before the enhancements described in this work (“DARC 1.0”), to its current performance (“DARC 2.0”). In this case, however, the ligand conformation drawn from the crystal structure was not included among those available during docking. Relative to the benchmark results shown in Fig 7, in this more challenging experiment “DARC 2.0” docks fewer ligands to within 2 Å RMSD of their positions in the crystal structure (now only 7 of 25 ligands).
(EPS)

S1 Table Small-molecule inhibitors bound to protein interaction sites. We compiled a set of 25 unique protein interaction sites for which a crystal structure has been solved in complex with a small-molecule inhibitor. This set is based upon our previous set of 21 complexes that were available at the time (drawn in part from the 2P2I [50] and TIMBAL [20] databases) [21], and we now add 4 additional examples have since become available. We did not include complexes with small molecule stabilizers, or complexes with small fragments or large peptide-like compounds. We only included one representative complex from each protein family; in cases where more than one suitable inhibitor-bound structure had been solved from a given family, we retained only the structure in complex with the most potent ligand. We also report here the number of conformers for each ligand used in these studies; conformers were generated using OMEGA (see Methods), and an average of 163 were used for the ligands in this set. Finally, we report the RMSD of the ligand conformer that is closest to the crystallographic ligand conformation. In the case of PDB ID 3IN7, for example, 300 conformers were generated but none were within 2 Å RMSD of the crystallographic conformation; inclusion of the crystallographic ligand conformation in the benchmark played a particularly important role in these cases.
(DOCX)
