# Supplementary figures and images for: DARC 2.0: Improved Docking and Virtual Screening at Protein Interaction Sites
Source: PLoS One. 2015 Jul 16;10(7):e0131612. doi: 10.1371/journal.pone.0131612 (PMC4504481; doi:10.1371/journal.pone.0131612)

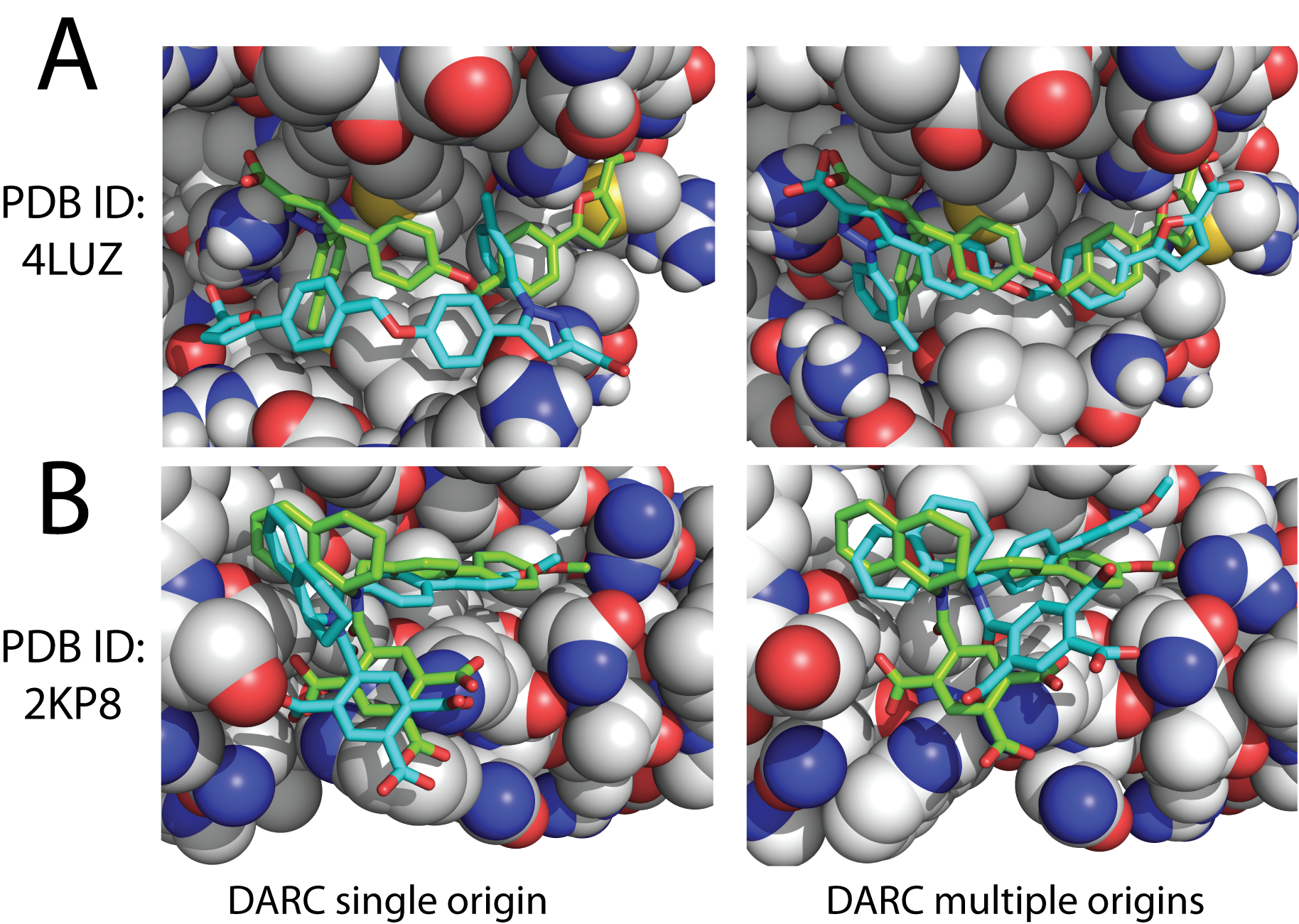

Supplement: S1 Fig — In all cases, the crystallographic ligand is shown in green, while the docked model is shown in cyan. (A) In this example (4LUZ, also shown in Fig 2C), the crystallographic ligand includes a ring facing directly into the protein (at the left side, in this perspective). The use of multiple origins captures the “walls” of this well, leading to a much-improved pose (RMSD goes from 11.7 Å to 1.9 Å). (B) Among examples for which performance was slightly deteriorated when using multiple origins (e.g. 2KP8, RMSD goes from 1.8 Å to 3.0 Å), the pockets were typically relatively flat and featureless; both docked poses appear to have equivalent shape complementarity, and the difference is presumably due simply to slight shifts in the relative ranking of these mis-docked poses when the origin is altered. (TIFF) [file pone.0131612.s002.tiff]

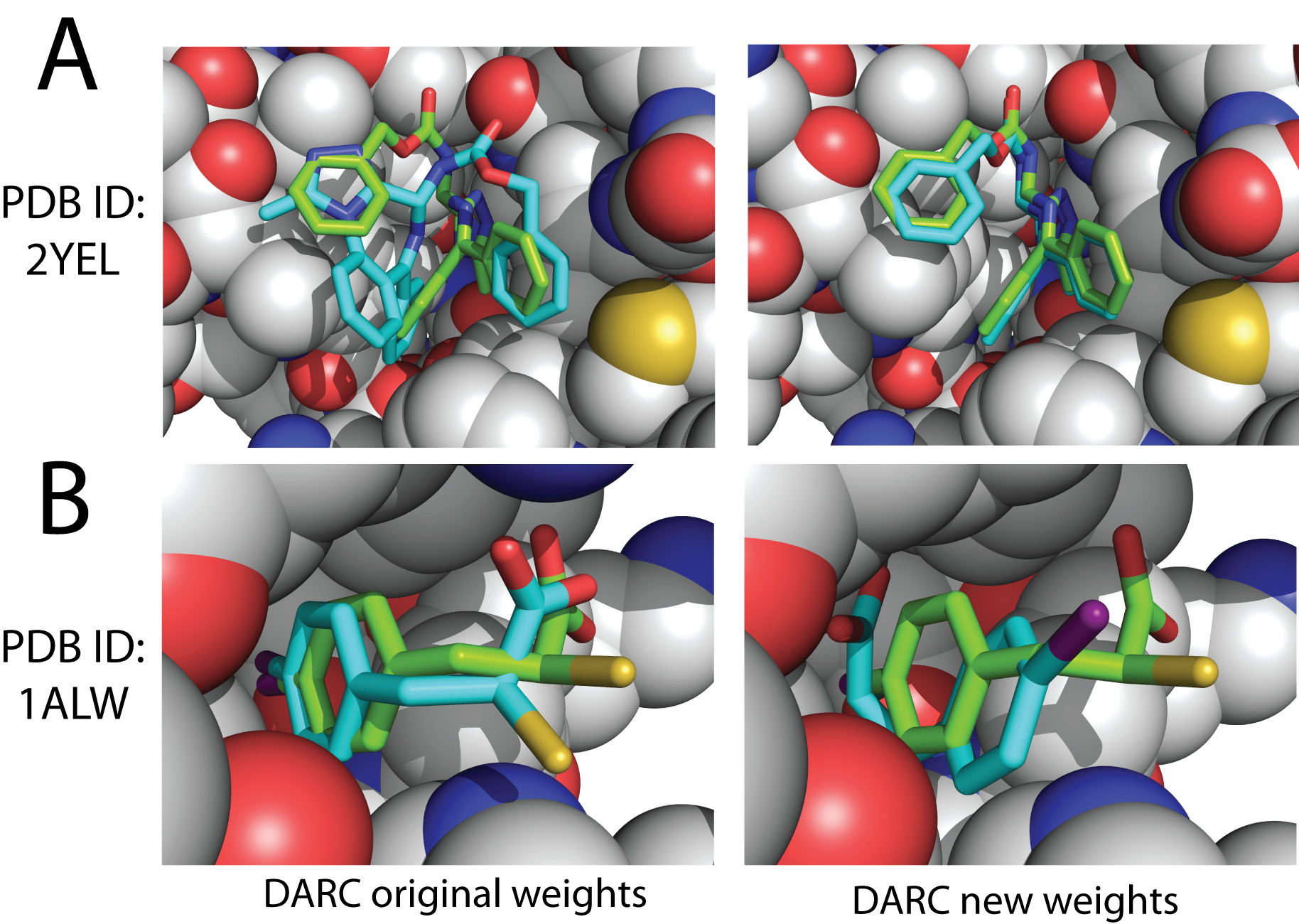

Supplement: S2 Fig — In all cases, the crystallographic ligand is shown in green, while the docked model is shown in cyan. (A) In this example (2YEL), the previous weight set may have insufficiently penalized underpacking at the protein-ligand interface. The new weights increase this penalty, leading to a much-improved pose (RMSD goes from 6.0 Å to 0.4 Å). (B) Among examples for which performance was slightly deteriorated when using the newer weight set (e.g. 1ALW, RMSD goes from 1.1 Å to 4.9 Å), the based for the diminished performance is not clear; both docked poses appear to have equivalent shape complementarity, and the difference is presumably due simply to slight shifts in the relative ranking of these mis-docked poses. (TIFF) [file pone.0131612.s003.tiff]

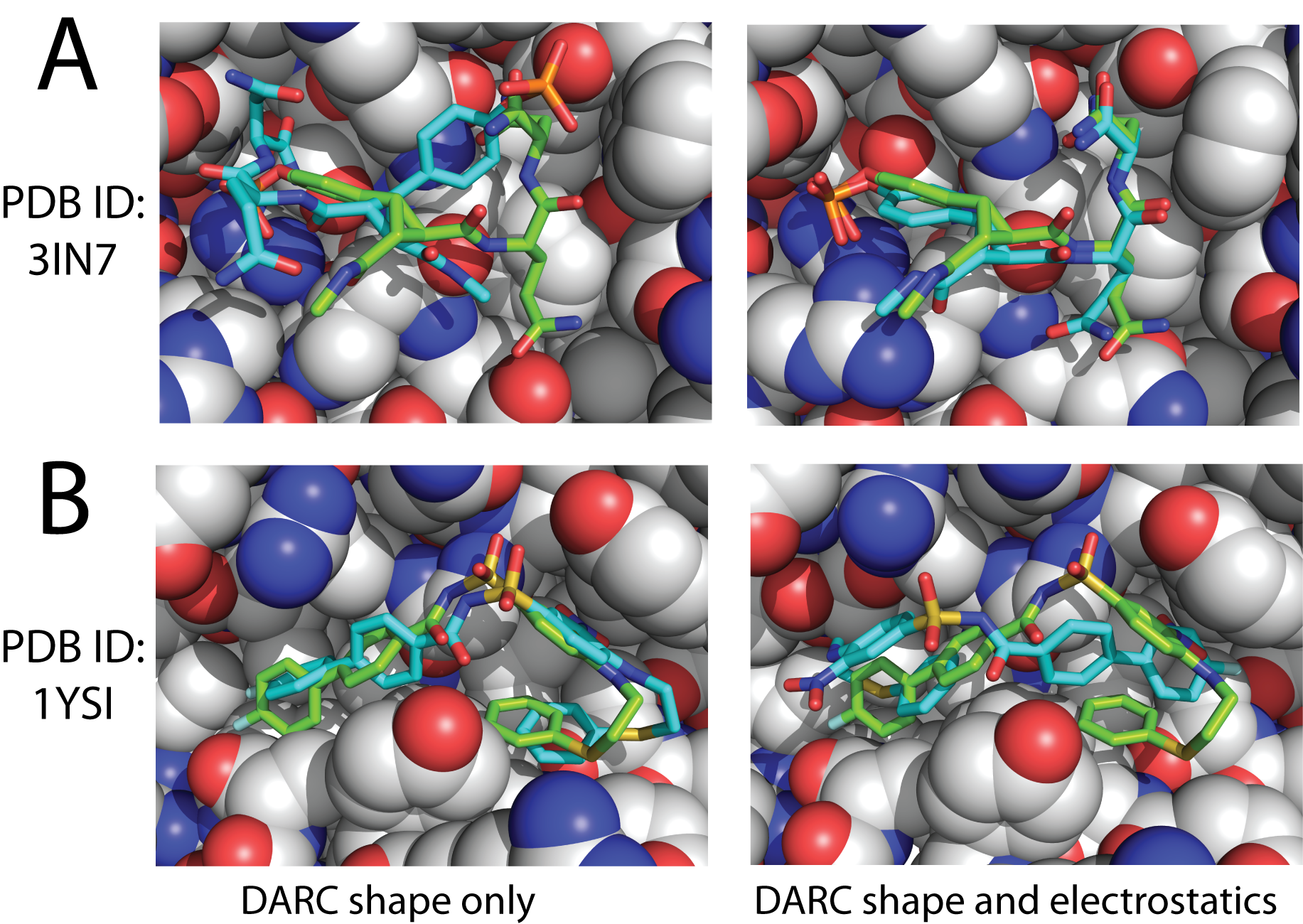

Supplement: S3 Fig — In all cases, the crystallographic ligand is shown in green, while the docked model is shown in cyan. (A) In this example (3IN7, RMSD goes from 8.7 Å to 0.8 Å), the crystallographic ligand binds to a flat region of the protein surface. Using shape alone, the correct pose is not clear; upon inclusion of the electrostatics term, the correct pose can be identified based on interactions around the (charged) phosphate group (at the left side of the native pose, in this perspective). (B) Among examples for which performance was slightly deteriorated when including electrostatics (e.g. 1YSI, RMSD goes from 2.7 Å to 11.0 Å), we find relatively non-polar protein surfaces with nearly symmetric binding pockets. While the sulfonamide group makes favorable electrostatic interactions in the native pose, the incorrect pose selected upon inclusion of electrostatics includes alternate (equally favorable) electrostatic interactions involving this group. (TIFF) [file pone.0131612.s004.tiff]
