# Supplementary material for: DARC 2.0: Improved Docking and Virtual Screening at Protein Interaction Sites
Source: PLoS One. 2015 Jul 16;10(7):e0131612. doi: 10.1371/journal.pone.0131612 (PMC4504481; doi:10.1371/journal.pone.0131612)
Supplement: S1 Table — We compiled a set of 25 unique protein interaction sites for which a crystal structure has been solved in complex with a small-molecule inhibitor. This set is based upon our previous set of 21 complexes that were available at the time (drawn in part from the 2P2I [50] and TIMBAL [20] databases) [21], and we now add 4 additional examples have since become available. We did not include complexes with small molecule stabilizers, or complexes with small fragments or large peptide-like compounds. We only included one representative complex from each protein family; in cases where more than one suitable inhibitor-bound structure had been solved from a given family, we retained only the structure in complex with the most potent ligand. We also report here the number of conformers for each ligand used in these studies; conformers were generated using OMEGA (see Methods), and an average of 163 were used for the ligands in this set. Finally, we report the RMSD of the ligand conformer that is closest to the crystallographic ligand conformation. In the case of PDB ID 3IN7, for example, 300 conformers were generated but none were within 2 Å RMSD of the crystallographic conformation; inclusion of the crystallographic ligand conformation in the benchmark played a particularly important role in these cases. (DOCX) [file pone.0131612.s006.docx]

# DARC 2.0: Improved docking and virtual screening

# at protein interaction sites

Ragul Gowthaman^1^, Sergey Lyskov^2^, and John Karanicolas^1,3*^

^1^ Center for Computational Biology, University of Kansas, 2030 Becker Dr., Lawrence, KS 66045

^2^ Department of Chemical and Biomolecular Engineering,

Johns Hopkins University, 3400 North Charles St., Baltimore, MD 21218

^3^Department of Molecular Biosciences, University of Kansas, 2030 Becker Dr., Lawrence, KS 66045

*To whom correspondence should be addressed. E-mail: [johnk@ku.edu](mailto:johnk@ku.edu), 785-864-8298

## Supplementary Tables

| **Protein** | **Ligand-bound PDB** | **Ligand conformers used** | **RMSD of “best” conformer (Å)** |
| --- | --- | --- | --- |
| calpain | 1ALW | 8 | 0.22 |
| calmodulin | 1CTR | 30 | 0.58 |
| IL-2 | 1PW6 | 300 | 1.80 |
| HPV E2 | 1R6N | 50 | 0.87 |
| XIAP-BIR3 | 1TFT | 175 | 1.21 |
| ZipA | 1Y2F | 300 | 0.61 |
| Bcl-xL | 1YSI | 300 | 1.10 |
| TNFα | 2AZ5 | 300 | 1.72 |
| HIV-gp41 | 2KP8 | 42 | 2.41 |
| integrin | 2VC2 | 300 | 0.63 |
| BRD4 | 2YEL | 250 | 0.54 |
| S100B | 3GK1 | 198 | 0.90 |
| Grb2-SH2 | 3IN7 | 300 | 2.40 |
| SHANK PDZ | 3O5N | 3 | 0.74 |
| WDR5 | 3UR4 | 300 | 0.95 |
| PCNA | 3VKX | 62 | 0.45 |
| VHL | 3ZRC | 300 | 0.76 |
| Plasminogen | 4CIK | 36 | 0.39 |
| HIV integrase | 4E1N | 1 | 3.05 |
| Mdm2 | 4ERF | 10 | 0.78 |
| clathrin | 4G55 | 300 | 1.75 |
| Menin | 4GQ4 | 175 | 0.68 |
| Cytohesin-2 | 4JMO | 7 | 1.72 |
| keap1 | 4L7D | 59 | 0.45 |
| RPA1 | 4LUZ | 264 | 1.39 |

**S1 Table: Small-molecule inhibitors bound to protein interaction sites.** We compiled a set of 25 unique protein interaction sites for which a crystal structure has been solved in complex with a small-molecule inhibitor. This set is based upon our previous set of 21 complexes that were available at the time (drawn in part from the 2P2I and TIMBAL databases), and we now add 4 additional examples have since become available. We did not include complexes with small molecule stabilizers, or complexes with small fragments or large peptide-like compounds. We only included one representative complex from each protein family; in cases where more than one suitable inhibitor-bound structure had been solved from a given family, we retained only the structure in complex with the most potent ligand. We also report here the number of conformers for each ligand used in these studies; conformers were generated using OMEGA (see *Methods*), and an average of 163 were used for the ligands in this set. Finally, we report the RMSD of the ligand conformer that is closest to the crystallographic ligand conformation. In the case of PDB ID 3IN7, for example, 300 conformers were generated but none were within 2 Å RMSD of the crystallographic conformation; inclusion of the crystallographic ligand conformation in the benchmark played a particularly important role in these cases.
